# Acoustic Sensors for Air and Surface Navigation Applications

**DOI:** 10.3390/s18020499

**Published:** 2018-02-07

**Authors:** Rohan Kapoor, Subramanian Ramasamy, Alessandro Gardi, Ron Van Schyndel, Roberto Sabatini

**Affiliations:** 1 School of Engineering, RMIT University, Aerospace Engineering and Aviation Discipline, Bundoora VIC 3083, Australia; rohan.kapoor@rmit.edu.au (R.K.); subramanian.ramasamy@rmit.edu.au (S.R.); alessandro.gardi@rmit.edu.au (A.G.); 2 School of Science, RMIT University, Computer Science and Information Technology Discipline, Melbourne 3000, Australia; ron.vanschyndel@rmit.edu.au

**Keywords:** acoustic sensors, intelligent transport systems, navigation, indoor navigation, ultrasonics, personal mobility, aerospace, ground vehicles

## Abstract

This paper presents the state-of-the-art and reviews the state-of-research of acoustic sensors used for a variety of navigation and guidance applications on air and surface vehicles. In particular, this paper focuses on echolocation, which is widely utilized in nature by certain mammals (e.g., cetaceans and bats). Although acoustic sensors have been extensively adopted in various engineering applications, their use in navigation and guidance systems is yet to be fully exploited. This technology has clear potential for applications in air and surface navigation/guidance for intelligent transport systems (ITS), especially considering air and surface operations indoors and in other environments where satellite positioning is not available. Propagation of sound in the atmosphere is discussed in detail, with all potential attenuation sources taken into account. The errors introduced in echolocation measurements due to Doppler, multipath and atmospheric effects are discussed, and an uncertainty analysis method is presented for ranging error budget prediction in acoustic navigation applications. Considering the design challenges associated with monostatic and multi-static sensor implementations and looking at the performance predictions for different possible configurations, acoustic sensors show clear promises in navigation, proximity sensing, as well as obstacle detection and tracking. The integration of acoustic sensors in multi-sensor navigation systems is also considered towards the end of the paper and a low Size, Weight and Power, and Cost (SWaP-C) sensor integration architecture is presented for possible introduction in air and surface navigation systems.

## 1. Introduction

The directionality of acoustic waves has been long used for localization by human beings. The term ‘echolocation’ was coined by Donald R. Griffin [1], where he discusses ship captains exploiting sound to ascertain the ship’s surroundings and avoid obstacles in low visibility environments. Acoustic sensors provide a low Size, Weight, and Power (SWaP) solution, which is low cost, scalable and robust. Moreover, acoustic sensors have the capability to provide high-resolution spatial information at short distance range. Radio-based localization techniques like Global Navigation Satellite Systems (GNSS) are prone to data degradations in dense urban environments and indoors [2]. On the other hand, electromagnetic techniques suffer from interference from other sources as well as metal structures. Optical navigation sensors are also still relatively expensive and their performance deteriorates in degraded visibility conditions as well as in environments consisting of optically transparent or opaque objects.

This paper discusses the fundamental principles and the technological considerations governing the use of sound waves for air and surface navigation applications, considering all known parasite effects. Before presenting the fundamental theory, Section 2 briefly reviews the role of echolocation in nature. In particular, this section delves into acoustic navigation systems employed by certain mammals, especially bats. Bats exhibit a very sophisticated acoustic echolocation system, which involves frequency, amplitude, and Pulse Repetition Frequency (PRF) modulation according to proximity to the prey or conspecifics [3,4,5]. These examples can greatly support the development of new navigation techniques, particularly when targeting emerging multi-Unmanned Aircraft System (UAS) formation flight and swarming. Section 3 addresses the theory governing the propagation of sound, particularly through the atmosphere, introducing all attenuation factors. Section 4 lists the ranging error sources and their modeling. Section 5 explains in detail the different system configurations: monostatic, multistatic, and a hybrid approach, which is a combination of the two. Monostatic as well as multistatic configurations are of particular interest as they can support a variety of applications in indoor navigation as well as personal mobility, including assistance for physically disabled. Section 6 details the state-of-the-art in acoustic sensors and also delves into the techniques developed for using acoustic sensors in navigation as well as personal mobility. In Section 7, the integration of acoustic sensors in the existing multi-sensor navigation systems for air and surface vehicles is discussed. In the final section, the paper sums up the findings, with evaluation of the scope of acoustic sensors in various navigation applications and future research trends.

## 2. Echolocation in Nature

Animals, especially mammals like bats and dolphins, use acoustic waves that vary in frequency, signal duration, and intensity, for navigation and tracking. Additionally, bats show the ability to detect and when needed, compensate for Doppler shift. Some interesting observations from bat echolocation are listed below:

- Bats can lower their call intensity as they approach strong reflective objects to prevent the echo sound pressure level from becoming too large. Bats can exhibit very high-resolution of target detection with time difference discriminations of 10–12 nanoseconds [3,6,7]. The duration of echolocation can vary considerably, with individual clicks being approximately ~50–100 μs long to constant frequency signals which are longer than 30 ms. Table 1 lists various bat species and their call type, based on their diet.
- As seen in the Table 1, the echolocation call can consist of a single frequency or multiple frequencies comprising a harmonic series. The pulse interval of the call also varies with proximity to the target. As bats approach their target, the repetition rate of their calls increases to get faster localisation updates. Also, the pulse interval of the call gives an indication of the maximum range from which bats can detect objects. Gleaning bats can passively listen to prey-generated sounds to localize their prey by interrupting echolocation or drastically reducing call intensity shortly before capturing prey [4].
- Big brown bats (*Eptesicus fuscus*) might roughly localise the position of its prey by listening to conspecific-generated echoes [5]. This is true for bottlenose dolphins (*Tursiops truncates*) as well [8].

Certain bats like Mexican free-tailed bat tend to increase their emission rate when flying in pairs. However, when flying in bigger groups, bats tend to decrease their emission rates, thereby reducing mutual interference [9]. This temporal modulation of emission in bats is similar to timing algorithms employed in electronic communication systems. These algorithms, also referred to as back-off algorithms, introduce probabilistic delays in resending packets lost due to interference [10]. 

Bats exhibit a variety of behaviour while coping with interference from environmental noise as well as both calls and echoes from nearby bats. While some species treat the presence of nearby conspecifics as any other source of noise or object in their field of view [13], certain species of bats like the free-tailed bats (*Molossidae*) compensate for interference by calling louder or by varying the frequency or duration of echolocation pulses [14]. 

Echolocation in bats refers to sensing of the environment based on Time of Arrival (TOA) of sound waves emitted by them. This helps bats navigate as well track their prey during the night. The strength of the received signal is indicative of the size of the target. Also, analysis of frequency spectrum of the echo gives an idea of the surface texture of the target. Most bat echolocation calls are ultrasonic, ranging from 20–200 kHz and the sound intensity can reach up to 130 dB. There have been attempts to investigate echolocation abilities in human beings as well, especially in visually impaired. In [15], the echolocation abilities of blind and sighted humans are reviewed, suggesting enhanced auditory abilities in visually impaired than normally sighted humans. The effect of prior visual experience on sound localisation in late blind individuals has been studied in detail in [16].

**Figure 1:**
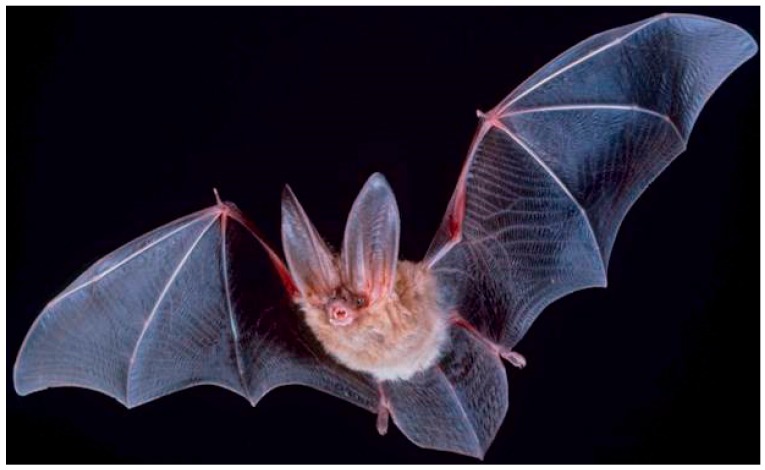
Bat pinnae of Townsend’s big-eared bat, *Corynohinus townsendi* [20].

There has been some research focus on analyzing the flight dynamics of bats and attempts made to emulate the same [17]. Bats use either their tongue or vocal chords to produce sonar signals [8]. Bats can vary the frequency, signal duration, signal intensity, harmonic composition and pulse interval according to their surroundings. Bats use narrowband signals for ranging of distant targets and broadband for localization. Some species of bats also account for Doppler shift by varying their call frequency [3,18]. Attempts have been made to develop biomimetic sonars inspired by bats’ external ears or pinnae (Figure 1), for localization and mapping, referred to as BatSLAM [19]. 

## 3. Sound Propagation

Acoustic waves are longitudinal waves that require a material medium to propagate. Fundamentally, sound can be defined as mechanical energy transmitted by pressure waves in a material medium. Acoustic waves are mechanical waves, i.e., they involve rapid to and fro displacements or vibrations of molecules in the medium. The velocity of sound in a medium ($c_{m}$) varies with the bulk modulus (B) and density (ρ) of the medium as shown in Equation (1). Sound travels faster in a medium with high bulk modulus or stiffness, like solids as compared to a medium with lower bulk modulus, like fluids:

(1) $$c_{m}=\sqrt{\frac{B}{\rho }}$$

The attenuation rate of sound waves varies with frequency, with higher frequencies attenuating at a faster rate. Attenuation can occur either due to reflection/scattering at interfaces or absorption [21]. However, higher frequencies, having short wavelengths, reflect strongly from small objects. Reflection from surfaces causes interference with the incident sound wave, which could be constructive or destructive. Interference depends upon the frequency of sound as well as the difference between the path length of direct and reflection paths [22]. Furthermore, the speed of sound in air varies with temperature, pressure, humidity, and wind, thereby affecting the propagation of sound. The generic equation for sound propagation can be given by:

(2) $$L_{p}\left(r\right)=L_{w}+\Sigma_{i}A_{i}$$

where $L_{p}\left(r\right)$: The sound pressure level at distance r from the source (dB); $L_{w}$: The sound power level of the source (dB); $A_{i}$: The combination of modifying factors that either attenuate or enhance the transmission of the sound energy as it propagates from source to receiver.

Acoustic sources have both far-field and near-field regions. Wavefronts produced by the sound source in near-field are not parallel and the intensity of the wave oscillates with range and the angle between source and receiver. However, in the far-field, wavefronts are nearly parallel, with intensity varying only with range to a centroid between sound sources, in accordance with the inverse squared rule. The near-field distance $r_{nf}$ is given by:

(3) $$r_{nf}=\frac{D^{2}}{\lambda }$$

where D is the equivalent aperture of the transmitter given by:

(4) $$D=\frac{3.2}{k\sin\left(\frac{\theta_{3\mathrm{dB}}}{2}\right)}$$

where k is the wave number and $\theta_{3\mathrm{dB}}$ is the half power beam angle. The wavefront for a sound source radiating equally in all directions is a sphere of radius r, whose intensity I from the source of power W is given by:

(5) $$I=\frac{W}{4\pi r^{2}}$$

Assuming a point source of sound in an unbounded homogenous atmosphere, the propagation of sound is affected by just two attenuating effects. While the first attenuation effect is geometric, which is solely dependent on the distance from the sound source, the second attenuating effect is the atmospheric absorption. Sound propagates due to the oscillation of air molecules about their mean position; with a higher frequency of sound leading to a higher rate of oscillation. This vibration of the air molecules leads to loss of energy through two dissipative mechanisms. While one of the mechanisms comprises of frictional losses, which includes both viscous action and heat conduction, the other mechanism involves the interaction of water vapour with the resonance of oxygen and nitrogen molecules. Hence, there are heat conduction losses, shear viscosity losses, and molecular relaxation losses [23]. 

### 3.1. Sound Attenuation in Atmosphere

However, in practical situations, the propagation of sound in the atmosphere is affected by additional factors like ground effects, attenuation due to finite barriers and buildings, reflections, wind, and temperature gradient effects, and atmospheric turbulence. The atmospheric sound attenuation factors are discussed in detail in Section 3.1.1, Section 3.1.2, Section 3.1.3, Section 3.1.4 and Section 3.1.5.

#### 3.1.1. Geometrical Divergence ($A_{div}$)

Geometrical divergence refers to the spherical spreading in the free field from a point sound source. The attenuating effect is the geometric attenuation, which results from the spreading of the radiated sound energy over a sphere of increasing diameter as the wavefront propagates away from the source. Equation (6) shows the relationship between the sound power level of the source, $L_{w}$, and sound pressure level, $L_{p}\left(r\right)$, at a distance r from that source. The variation of sound pressure level with distance from the source is shown in Equation (7). Unlike atmospheric attenuation, geometric attenuation is independent of the frequency of the propagating sound wave. From Equation (7), it can be inferred that sound intensity or sound pressure level, $L_{p}$ decreases by 6 dB per doubling of distance away from the source:

(6) $$L_{p}\left(r\right)=L_{w}+10\log\left[\frac{1}{4\pi r^{2}}\right]$$

(7) $$L_{p}\left(r_{2}\right)=L_{p}\left(r_{1}\right)+20\log\left[\frac{r_{1}}{r_{2}}\right]$$

The geometrical divergence, in dB, is given by:

(8) $$A_{div}=\left[20\log\left(\frac{d}{d_{0}}\right)+11\right]$$

where d is the distance from the sound source to receiver (m) and $d_{0}$ is the reference distance which is 1 m from an omnidirectional point sound source. 

#### 3.1.2. Atmospheric Absorption ($A_{\mathrm{atm}}$)

Air absorption becomes significant at higher frequencies and at long ranges, thereby acting as a low-pass filter at long range. The pressure of a planar sound wave at a distance x from a point of pressure $P_{0}$ is given by:

(9) $$P=P_{0}e^{\frac{-\alpha x}{2}}$$

The attenuation coefficient, α, for air absorption depends on frequency, humidity, temperature, and pressure, with its value being calculated using Equations (10)–(12) [24].

(10) $$\alpha =f^{2}\left[\left(\frac{1.84\times 10^{-11}}{{\left(\frac{T_{0}}{T}\right)}^{1/2}\frac{p_{s}}{p_{0}}}\right)+{\left(\frac{T_{0}}{T}\right)}^{2.5}\times \left(\frac{0.10680e^{-3352/T}f_{r,N}}{f^{2}+f_{r,N}^{2}}+\frac{0.01278e^{-2239.1/T}f_{r,O}}{f^{2}+f_{r,O}^{2}}\right)\right]$$

where f is the frequency, T is the absolute temperature of the atmosphere in kelvins, $T_{0}$ is the reference value of T (293.15 K) and $f_{r,N}$ and $f_{r,O}$ are relaxation frequencies associated with the vibration of nitrogen and oxygen molecules, respectively, and are given by:

(11) $$f_{r,N}=\frac{p_{s}}{p_{0}}{\left(\frac{T_{0}}{T}\right)}^{1/2}\left(9+280He^{-4.17\left[{\left(T_{0}/T\right)}^{\frac{1}{3}}-1\right]}\right)$$

(12) $$f_{r,O}=\frac{p_{s}}{p_{0}}\left(24.0+4.04\times 10^{4}H\frac{0.02+H}{0.391+H}\right)$$

where $p_{s}$ is local atmospheric pressure, $p_{0}$ is the reference atmospheric pressure (101,325 Pa) and H is the percentage molar concentration of water vapour in the atmosphere which is given by:

(13) $$H=\frac{\rho_{sat}r_{h}p_{0}}{p_{s}}$$

where $r_{h}$ is the relative humidity and $\rho_{sat}$ is given by:

(14) $$\rho_{sat}=10^{C_{sat}}$$

where $C_{sat}$ is given by:

(15) $$C_{sat}=-6.8346{\left(\frac{T_{0}}{T}\right)}^{1.261}+4.6151$$

Similarly, $\rho_{sat}$ can also be written as [25,26]:

(16) $$\rho_{sat}=1322.8{\left(\frac{r_{h}}{T}\right)}^{\left[\frac{25.22\left(T-273.15\right)}{T}-5.31\ln\left(\frac{T}{273.15}\right)\right]}$$

**Figure 2:**
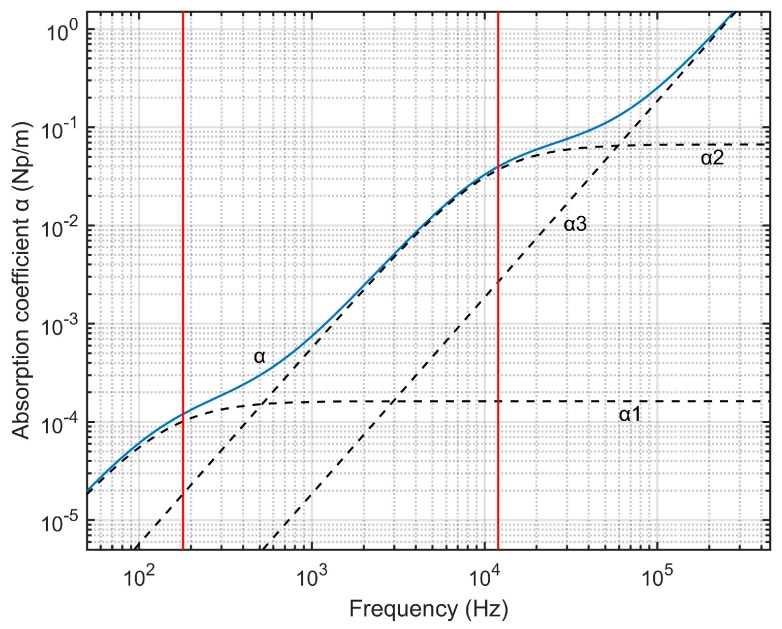
Attenuation of sound in air as a function of frequency (log-log plot).

Figure 2 shows the variation of absorption coefficient α with the frequency of sound at 293.15 K, one atmospheric pressure, 20% relative humidity and H being 4.7 × 10^−3^. As there are two relaxation frequencies associated with oxygen and nitrogen, the frequency dependence of the attenuation coefficient for sound in the air has three distinct regions. At very low frequencies, where the sound frequency is much lower than that associated with nitrogen molecules, the attenuation is dominated by vibrational relaxation of nitrogen molecules ($\alpha_{1}$). The frequency dependence is quadratic with an apparent bulk viscosity associated with the nitrogen relaxation. In the intermediate region, the frequency is substantially larger than that associated with nitrogen relaxation, but still substantially less than that associated with oxygen relaxation, with quadratic frequency dependence, smaller coefficient and apparent bulk viscosity that is associated with oxygen relaxation ($\alpha_{2}$). In the higher frequency region, there is quadratic dependence again, although with an even smaller coefficient, and with the intrinsic bulk viscosity associated with molecular rotation ($\alpha_{3}$) [24]. 

Having calculated the absorption coefficient for a given temperature, pressure, relative humidity and percentage molar concentration of water vapor in the atmosphere, the attenuation of sound due to atmospheric absorption, during propagation through a distance d (m) is given by:

(17) $$A_{\mathrm{atm}}=\alpha d/1000$$

#### 3.1.3. Ground Effect ($A_{gr}$)

**Figure 3:**
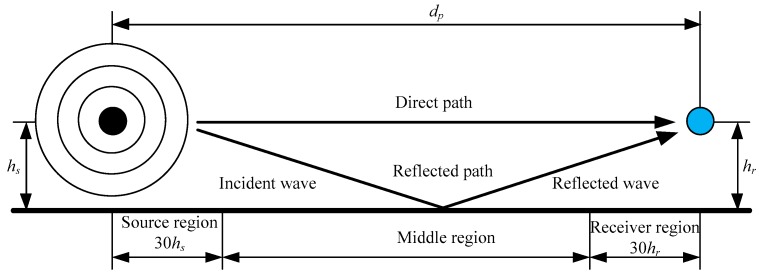
Effect of ground on the received sound pressure level.

Adding the effect of a bounding ground plane to the sound propagation model allows for sound to propagate directly from source to receiver as well as through secondary propagation path resulting from a reflection off the ground plane as shown in Figure 3. This secondary propagation path can result in interference effects between the direct and reflected waves at the receiver. The interference effect can be constructive or destructive, depending on the relative amplitudes and phase of the direct and reflected waves. The relationship between the direct and reflected waves depends on a variety of factors including the difference between the direct and reflected path lengths, which is a function of source and receiver separation distance (dp) as well as their height above the ground ($h_{s}$ and $h_{r}$), the wavelength of sound and reflective properties of the ground which can cause variations in the phase and amplitude of the reflected sound wave.

Based on the acoustical properties of ground surfaces, they are classified into three types based on the ground factor (G). Hard ground, which has a G of 0, includes concrete, paving, water, ice and all other low porosity ground surfaces. On the other hand, porous ground, which has a G of 1, consists of grass, trees, foliage and all other ground surfaces which are suitable for the growth of vegetation. Surfaces which are a combination of both hard and porous grounds, i.e., having a value of G ranging from 0 to 1, where G represents the fraction of the ground surface that is porous, are known as mixed ground. The total ground attenuation is obtained by summing up the attenuation $A_{s}$ for the source region specified by the ground factor $G_{s}$, $A_{m}$ for the middle region specified by the ground factor $G_{m}$, and $A_{r}$ for the receiver region specified by the ground factor $G_{r}$, as shown in Equation (18):

(18) $$A_{gr}=A_{s}+A_{m}+A_{r}$$

#### 3.1.4. Screening ($A_{bar}$)

**Figure 4:**
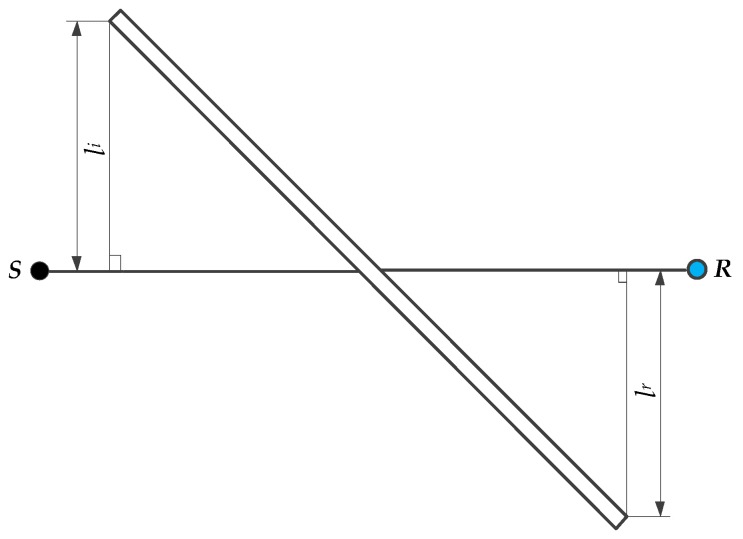
Obstacle between the source (*S*) and the receiver (*R*).

**Figure 5:**
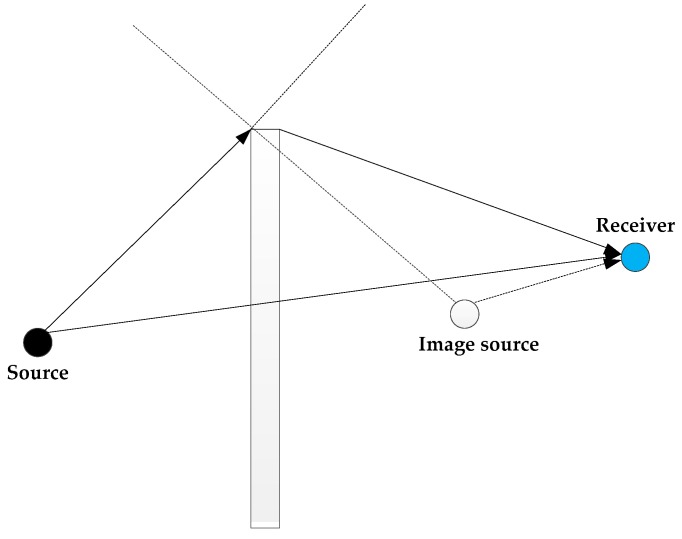
Diffraction of sound by a thin barrier.

An object shall be considered as a screening obstacle (Figure 4) if it meets the following requirements:

- The object has a surface density of at least 10 kg/m^2^;
- The surface of the object is closed without cracks or gaps;
- The horizontal dimension of the object normal to the source-receiver line ($l_{i}+l_{r}$) is larger than the acoustic wavelength λ at the nominal midband frequency for the octave band of interest, i.e., $l_{i}+l_{r}>\lambda$ (Figure 5).

The barrier diffraction could be either single diffraction in case of thin barriers (Figure 5) or double diffraction in thick barriers. In case of more than two barriers, the barrier attenuation can be approximated to be a case of double diffraction, by choosing the two most effective barriers, neglecting the effect of the others. The effect diffraction (in dB) for downward sound propagation over the top edge and the vertical edge, respectively, is given by Equations (19) and (20) [27]. $D_{Z}$ is the barrier attenuation for each octave band and $A_{gr}$ is the ground attenuation in the absence of the barrier, as described in Section 3.1.3.

(19) $$A_{bar}=D_{Z}-A_{gr}>0$$

(20) $$A_{bar}=D_{Z}>0$$

#### 3.1.5. Wind and Temperature Gradient Effects

As a result of uneven heating of the Earth’s surface, the atmosphere is constantly in motion. The turbulent flow of air across the rough solid surface of the Earth generates a boundary layer. The lower part of the meteorological boundary layer, called the surface layer, extends over 50–100 m in typical daytime conditions [24]. Turbulent fluxes vary by less than 10% of their magnitude in the surface layer, but the wind speed and temperature gradients are the largest. Turbulence can be modelled as a series of moving eddies with a distribution of sizes. Various turbulence models like Gaussian, Von Kármán, and Kolmogorov are used in atmospheric acoustics [28,29,30]. It has been shown that turbulence effects decrease with increase in elevation of sound sources from the ground [31]. 

As the temperature decreases with height, in the absence of wind, this causes sound waves to bend, or refract, upwards. Wind velocity either adds or subtracts from the velocity of sound, depending upon whether the source is upwind or downwind of the receiver, height above ground and temperature inversions. Wind effects tend to dominate over temperature effects when both are present. The general relationship between the speed of sound profile c(z), the temperature profile T(z) and wind speed profile u(z) in the direction of sound propagation, for a height z, is given by [32]:

(21) $$c\left(z\right)=c\left(0\right)\sqrt{\frac{T\left(z\right)+273.15}{273.15}}+u\left(z\right)$$

#### 3.1.6. Other Sound Attenuation Factors

Various standardised techniques for measuring the attenuation of sound outdoors due to atmospheric absorption effects have been developed in ISO 9613-1 and ISO 9613-2 [27,33]. This includes attenuation of sound due to miscellaneous effects like foliage, housing or industrial sites. The analytical models presented rely on the values of various atmospheric parameters like temperature, pressure, relative humidity, wind speed and time of the day. Besides, fog and precipitation can also affect the attenuation of sound [34]. Experiments show that precipitation affects the temperature variation, hence indirectly affecting sound attenuation outdoors [31]. Hence, to summarize, the attenuation of sound in atmosphere can be given by:

(22) $$A=A_{div}+A_{\mathrm{atm}}+A_{gr}+A_{bar}+A_{misc}$$

where $A_{misc}$ is the sound attenuation due to other miscellaneous effects like wind and temperature gradient effects, precipitation, foliage, and housing or industrial sites.

## 4. Echolocation Errors

Table 2 gives the different ranging parameters involved in the design of the acoustic sensors. The ranging equation is given by Equation (23), where $R_{m}$ is the measured range, $R_{a}$ is the actual range from the transmitter $\left(x_{T},y_{T},z_{T}\right)$ to the receiver $\left(x_{R},y_{R},z_{R}\right)$ and ε is the error in the measured range. The error term ε comprises mainly of error due to multipath ($\varepsilon_{Mp}$), Doppler shift ($\varepsilon_{Ds}$) and atmospheric effects ($\varepsilon_{Atm}$):

(23) $$R_{m}=R_{a}+\varepsilon$$

where:

(24) $$R_{a}=\sqrt{{\left(x_{T}-x_{R}\right)}^{2}+{\left(y_{T}-y_{R}\right)}^{2}+{\left(z_{T}-z_{R}\right)}^{2}}$$

(25) $$\varepsilon =\varepsilon_{Ds}+\varepsilon_{Mp}+\varepsilon_{Atm}$$

### 4.1. Doppler Effect

**Figure 6:**
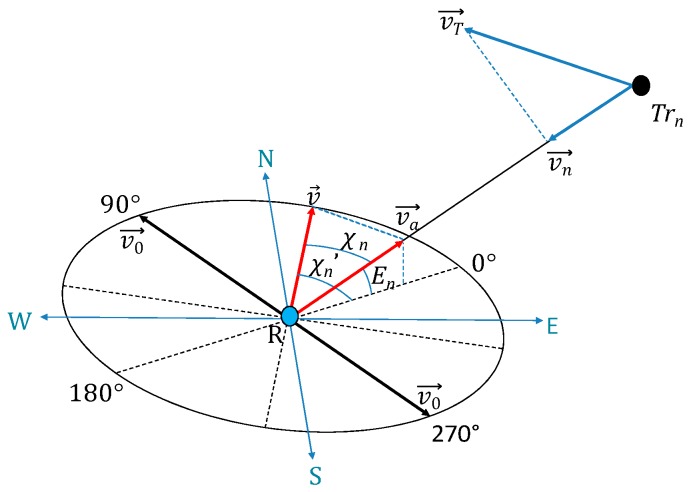
Reference geometry for Doppler shift analysis.

The Doppler effect is caused by the perceived change in sound frequency due to relative motion between sound source and receiver. Figure 6 shows the elevation angle ($E_{n}$) of the *n*th transmitter ($Tr_{n}$) to the receiver (R) as well as the relative bearing ($\chi_{n}$), the tangential velocity of the transmitter ($\vec{v_{T}}$) and the azimuth of the Line of Sight (LOS) projection ($\chi_{n}\prime$). $\vec{v_{0}}$ is the velocity of the receiver and the case of $\vec{v_{0}}=\vec{v}$ results in a null Doppler shift as no component of receiver velocity vector is in the direction of LOS to the transmitter. As is evident from Equation (26), the Doppler shift is inversely proportional to both elevation and azimuth angle. The pitch increases as the relative distance between the sound source and receiver decreases. The change in observed frequency of sound is given as:

(26) $$\Delta f_{n}=f\left(\frac{\left|\vec{v_{n}}\right|\mp \left|\vec{v_{a}}\right|}{c}\right)\frac{\cos\chi_{n}\prime }{\sin E_{n}}$$

where $\vec{v_{n}}$ = *n*th transmitter velocity component along the LOS; $\vec{v_{a}}$ = receiver velocity projection along the LOS; c = speed of sound $({\mathrm{ms}}^{-1}$); f = sound frequency (Hz); $E_{n}$ = elevation angle of the *n*th transmitter; $\chi_{n}\prime$ = azimuth of the LOS projection.

**Figure 7:**
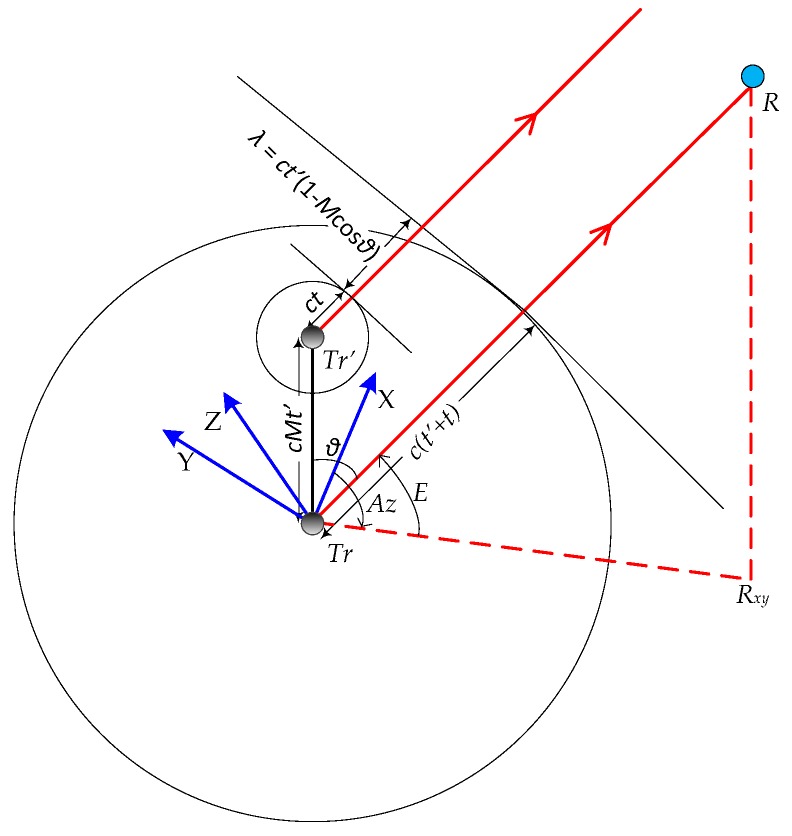
Doppler shift sound field.

Considering a simplified case where transmitter motion and LOS from the transmitter to the receiver are coplanar, the sound field at times t and (t+t′) for a moving sound source (Tr) which has moved to a new point (Tr′) in time t′, is shown in Figure 7. 

The moving sound source emits crests every t′ units of time. The Doppler-shifted frequency $f^{\prime }$ from a moving sound source emitting frequency $f_{s}$ is given by:

(27) $$f^{\prime }=\frac{f_{s}}{1-M\cos\;\theta }$$

where M is the Mach number for the sound source and θ is the direction of the receiver to the sound source at the time (t+t′).

Assuming no relative motion between transmitter-receiver and the speed of the transmitter being Mc, if it takes time t for sound to reach the receiver from the transmitter, the error in range due to Doppler shift is given by:

(28) $$\varepsilon_{Ds}=Mct\cos\;\theta$$

where θ is the direction of receiver motion relative to the LOS between the transmitter and the receiver.

### 4.2. Multipath 

An important property of a medium that influences the strength or amplitude of reflected waves is acoustic impedance. Acoustic impedance can be defined as the product of the density of the medium (ρ) and speed of sound ($c_{m}$) in the medium:

(29) $$Z=\rho c_{m}$$

Acoustic impedance gives a measure of the sound transmitted and reflected back at the interface of two mediums. The ratio of the reflected pressure amplitude, $P_{r}$, to the incident pressure amplitude, $P_{i}$, called amplitude reflection coefficient, is given by:

(30) $$R_{P}=\frac{P_{r}}{P_{i}}=\frac{Z_{2}-Z_{1}}{Z_{2}+Z_{1}}$$

**Figure 8:**
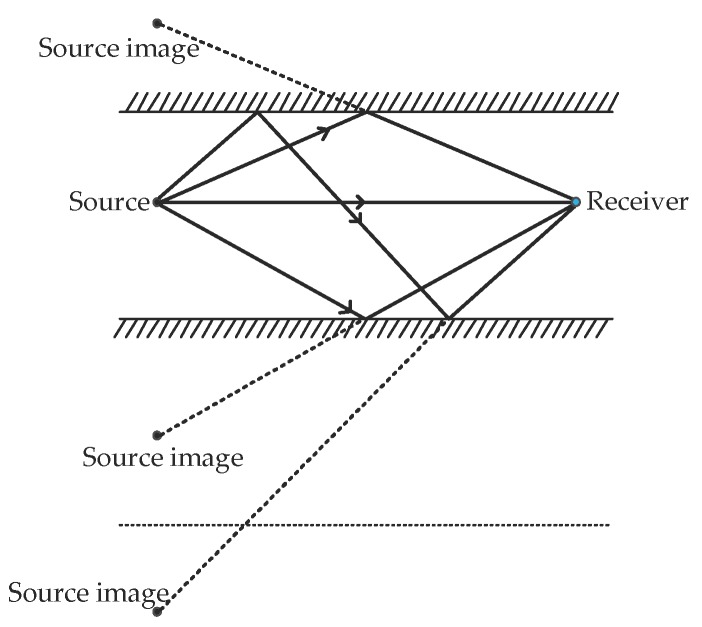
Multipath.

Assuming a homogenous medium, sound propagation, especially at high frequencies, can be assumed to be a straight line from the source to the receiver [35]. Assuming a range independent geometry for a homogenous medium, the sound waves are subject to multiple reflections, as shown in Figure 8. As most of the reflecting surfaces are irregular, sound waves experience a significant amount of scattering or diffraction on reflection. 

**Figure 9:**
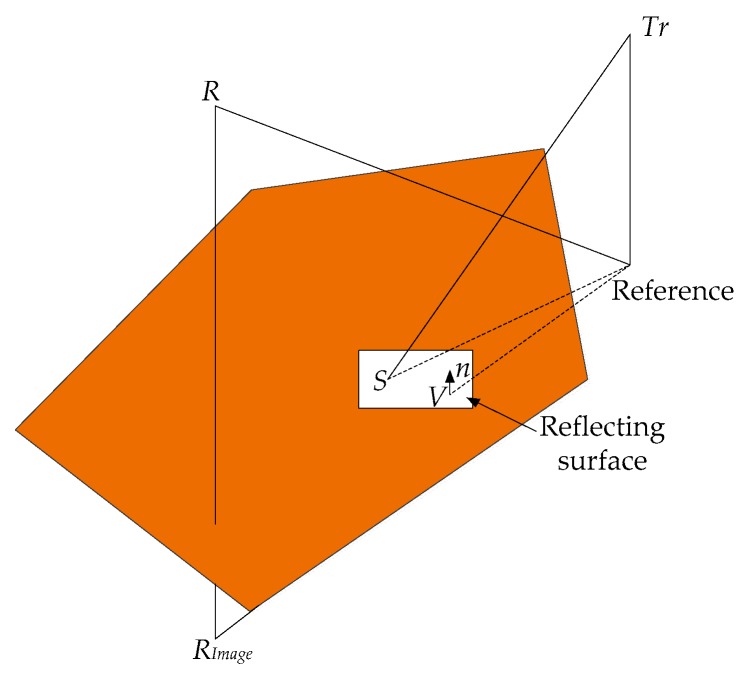
Geometric reflection model.

Using ray-tracing [36,37], the reflection point S and the defined point V, as shown in Figure 9, should satisfy the equation:

(31) (S−V)×n=0

where sound waves are emitted from point Tr to the receiver location R after reflection at point S. V is defined as a point on the reflecting surface and n is a unit vector normal to that surface. The line equation connecting Tr and $R_{image}$ is given by:

(32) $$S=Tr+m\left(R_{image}-T\right)$$

where m is a parameter between 0 and 1. Combining Equations (31) and (32):

(33) $$S=Tr+\frac{n\times V-n\times Tr}{n\times \left(R_{image}-Tr\right)}\left(R_{image}-Tr\right)$$

Assuming specular reflection, the extra path length $L_{mS}$ is given by:

(34) $$L_{mS}=\left|Tr-S\right|+\left|R-S\right|-\left|Tr-R\right|$$

The starting point for performing ray-tracing of acoustic waves is Helmholtz equation, which can be written in Cartesian coordinates x=(x,y,z) as:

(35) $$\nabla^{2}p+\frac{\omega^{2}}{c^{2}\left(x\right)}p=-\delta \left(x-x_{0}\right)$$

where p is the total pressure, ω is the angular frequency of the source located at $x_{0}$. A solution of the Helmholtz equation, which is called the ray series, is used to obtain the ray equations [38]. The ranging error due to multipath is given by:

(36) $$\varepsilon_{Mp}=\overset{N}{\underset{i=1}{\sum }}\left|Tr_{i}-S_{i}\right|+\left|R_{i}-S_{i}\right|-\left|Tr_{i}-R_{i}\right|$$

where: $Tr_{i}$ = location of *i*th acoustic transmitter; $S_{i}$ = *i*th reflection point; $R_{i}$ = location of *i*th acoustic receiver; N = number of reflecting surfaces.

### 4.3. Atmospheric Effects

In the “International Standard Atmosphere” (ISA), the troposphere extends up to 11 km. The temperature gradient in the troposphere can be assumed to be constant. Following this assumption, the ranging error due to atmospheric effects is given by:

(37) $$\varepsilon_{Atm}=\left[\left(c^{\prime }t+c_{w}t\right)-ct\right]$$

where $c_{w}$ is the variation of the speed of sound due to the wind. The variation of the speed of sound due to temperature [39] is given by:

(38) $$c^{\prime }=c_{0}+\frac{dc}{dH}H$$

where: $c_{0}$ = speed of sound at sea-level; H = height above sea-level; $\frac{dc}{dH}$ = $\frac{\lambda c_{0}}{2T_{0}}$ is the gradient of the speed of sound, where $T_{0}$ is the sea-level temperature (K) and $\lambda =\frac{dT}{dH}$ is the variation of temperature with height. The variation of the speed of sound due to wind is given by:

(39) $$c_{w}=\frac{c_{r}}{cos\delta }+v_{w}$$

where: $c_{r}$ = speed of sound relative to air; δ = angle of wavefront normal with the horizontal; $v_{w}$ = horizontal wind velocity.

The magnitude of the horizontal wind velocity near the Earth’s surface is predominantly determined by the prevailing horizontal pressure gradient in the atmosphere and the surface friction [39]. The surface friction arises from the relative motion between air and the ground surface and has to be accounted for heights up to 1000 m. 

### 4.4. Ranging Error Analysis

The range measured by acoustic sensors can be written as:

(40) $$R=c_{0}t+Mct\cos\;\theta +\overset{N}{\underset{i=1}{\sum }}\left|Tr_{i}-S_{i}\right|+\left|R_{i}-S_{i}\right|-\left|Tr_{i}-R_{i}\right|+\int_{0}^{t}(\frac{dc}{dH}R\cos\theta +v_{w}\cos\delta )dt$$

(41) $$=c_{0}t+Mct\cos\;\theta +\overset{N}{\underset{i=1}{\sum }}\left|Tr_{i}-S_{i}\right|+\left|R_{i}-S_{i}\right|-\left|Tr_{i}-R_{i}\right|+Rt\cos\theta \frac{dc}{dH}+v_{w}t\cos\delta$$

where t is the time of flight. R can be rewritten as:

(42) $$R=\frac{c_{0}t+Mct\cos\;\theta +\sum_{i=1}^{N}\left[\left|Tr_{i}-S_{i}\right|+\left|R_{i}-S_{i}\right|-\left|Tr_{i}-R_{i}\right|\right]+v_{w}t\cos\delta }{1-t\cos\theta \frac{dc}{dH}}$$

(43) $$=\frac{c_{0}t+Mct\cos\;\theta +\sum_{i=1}^{N}\left[\left|Tr_{i}-S_{i}\right|+\left|R_{i}-S_{i}\right|-\left|Tr_{i}-R_{i}\right|\right]+v_{w}t\cos\delta }{1-t\cos\theta \frac{c_{0}\lambda }{2T_{0}}}$$

The uncertainty in range measurement can be obtained by calculating the deviation in range measurement error from all error sources. The cumulative deviation of ranging error is given by:

(44) $$\sigma_{R}=\sqrt{\begin{array}{l} {\left(\frac{\partial R}{\partial c_{0}}\right)}^{2}\sigma_{c}{{}_{{}_{0}}}^{2}+{\left(\frac{\partial R}{\partial t}\right)}^{2}\sigma_{t}{}^{2}+{\left(\frac{\partial R}{\partial \theta }\right)}^{2}\sigma_{\theta }{}^{2}+{\left(\frac{\partial R}{\partial T_{0}}\right)}^{2}\sigma_{T_{0}}{}^{2}+{\left(\frac{\partial R}{\partial \lambda }\right)}^{2}\sigma_{\lambda }{}^{2}+{\left(\frac{\partial R}{\partial M}\right)}^{2}\sigma_{M}{}^{2}+\cdots \\ {\left(\frac{\partial R}{\partial c}\right)}^{2}\sigma_{c}{}^{2}+{\left(\frac{\partial R}{\partial Tr_{i}}\right)}^{2}\sigma_{Tr_{i}}{}^{2}+{\left(\frac{\partial R}{\partial S_{i}}\right)}^{2}\sigma_{S_{i}}{}^{2}+{\left(\frac{\partial R}{\partial R_{i}}\right)}^{2}\sigma_{R_{i}}{}^{2}+{\left(\frac{\partial R}{\partial \nu_{w}}\right)}^{2}\sigma_{\nu_{w}}{}^{2}+{\left(\frac{\partial R}{\partial \delta }\right)}^{2}\sigma_{\delta }{}^{2} \end{array}}$$

where:

(45) $$\frac{\partial R}{\partial c_{0}}=\left(\frac{t\cos\theta \lambda \left(c_{0}t+Mct\cos\theta +\sum_{i=1}^{N}\left|Tr_{i}-S_{i}\right|+\left|R_{i}-S_{i}\right|-\left|Tr_{i}-R_{i}\right|+v_{w}t\cos\delta \right)}{2T_{0}{\left(1-t\cos\theta \frac{c_{0}\lambda }{2T_{0}}\right)}^{2}}+\frac{t}{\left(1-t\cos\theta \frac{c_{0}\lambda }{2T_{0}}\right)}\right)$$

(46) $$\frac{\partial R}{\partial t}=\left(\frac{2T_{0}c_{0}\lambda \cos\theta \left(c_{0}t+Mct\cos\;\theta +\sum_{i=1}^{N}\left|Tr_{i}-S_{i}\right|+\left|R_{i}-S_{i}\right|-\left|Tr_{i}-R_{i}\right|+v_{w}t\cos\delta \right)}{{\left(2T_{0}-c_{0}t\lambda \cos\theta \right)}^{2}}+\frac{c_{0}+Mc\cos\;\theta +v_{w}\cos\delta }{1-t\cos\theta \frac{c_{0}\lambda }{2T_{0}}}\right)$$

(47) $$\frac{\partial R}{\partial \theta }=\left(\frac{-2T_{0}tc_{0}\lambda \sin\theta \left(c_{0}t+Mct\cos\;\theta +\sum_{i=1}^{N}\left|Tr_{i}-S_{i}\right|+\left|R_{i}-S_{i}\right|-\left|Tr_{i}-R_{i}\right|+v_{w}t\cos\delta \right)}{{\left(2T_{0}-c_{0}t\lambda \cos\theta \right)}^{2}}-\frac{Mct\sin\;\theta }{1-t\cos\theta \frac{c_{0}\lambda }{2T_{0}}}\right)$$

(48) $$\frac{\partial R}{\partial T_{0}}=\left(-\frac{2c_{0}t\lambda \cos\theta \left(c_{0}t+Mct\cos\;\theta +\sum_{i=1}^{N}\left|Tr_{i}-S_{i}\right|+\left|R_{i}-S_{i}\right|-\left|Tr_{i}-R_{i}\right|+v_{w}t\cos\delta \right)}{{\left(2T_{0}-c_{0}t\lambda \cos\theta \right)}^{2}}\right)$$

(49) $$\frac{\partial R}{\partial \lambda }=\left(\frac{2T_{0}c_{0}t\cos\theta \left(c_{0}t+Mct\cos\;\theta +\sum_{i=1}^{N}\left|Tr_{i}-S_{i}\right|+\left|R_{i}-S_{i}\right|-\left|Tr_{i}-R_{i}\right|+v_{w}t\cos\delta \right)}{{\left(2T_{0}-c_{0}t\lambda \cos\theta \right)}^{2}}\right)$$

(50) $$\frac{\partial R}{\partial M}=\left(\frac{ct\cos\theta }{1-t\cos\theta \frac{c_{0}\lambda }{2T_{0}}}\right)$$

(51) $$\frac{\partial R}{\partial c}=\left(\frac{Mt\cos\theta }{1-t\cos\theta \frac{c_{0}\lambda }{2T_{0}}}\right)$$

(52) $$\frac{\partial R}{\partial S_{i}}=\left(\frac{2}{1-t\cos\theta \frac{c_{0}\lambda }{2T_{0}}}\right)$$

(53) $$\frac{\partial R}{\partial Tr_{i}}=\frac{\partial R}{\partial R_{i}}=0$$

(54) $$\frac{\partial R}{\partial v_{w}}=\left(\frac{t\cos\delta }{1-t\cos\theta \frac{c_{0}\lambda }{2T_{0}}}\right)$$

(55) $$\frac{\partial R}{\partial \delta }=\left(\frac{v_{w}t\sin\delta }{1-t\cos\theta \frac{c_{0}\lambda }{2T_{0}}}\right)$$

The error budgeting has been numerically validated in a case study, taking realistic values of variables, as shown in Table 3. This analysis gives an error of 0.24 m for a range of 10 m, with more than 95% of the error being due to multipath. However, in real life situations, the hardware limitations associated with the practical realization of acoustic sensors can introduce additional errors, which have to be considered as well in the overall error budgeting.

## 5. Sensor Arrangements

### 5.1. Monostatic Approach

A major limitation of the multistatic system is that it works in predetermined environments only. To overcome this limitation, a monostatic approach can be applied for obstacle detection and tracking. The generalized SONAR (Sound Navigation and Ranging) equation is given by:

(56) SNL=SL−2TL+TS−(NL−DI)

where: SNL = signal-to-noise ratio of the returning echo; SL = source sound level; 2TL = two-way transmission losses; TS = target strength; NL = noise level; DI = directivity index.

This approach utilizes a collocated transceiver. The transmission losses are a function of spherical spreading and atmospheric attenuation. The basic range equation for the monostatic approach is:

(57) $$\rho_{k}{}^{t}\left(t_{k}\right)=\left(t_{k}-t^{t}\right)c/2$$

where $\rho_{k}{}^{t}$ is the actual measurement, $t_{k}$ is the nominal time of reception, $t^{t}$ is the nominal time of emission, and c is the speed of sound. 

### 5.2. Multistatic Approach

**Figure 10:**
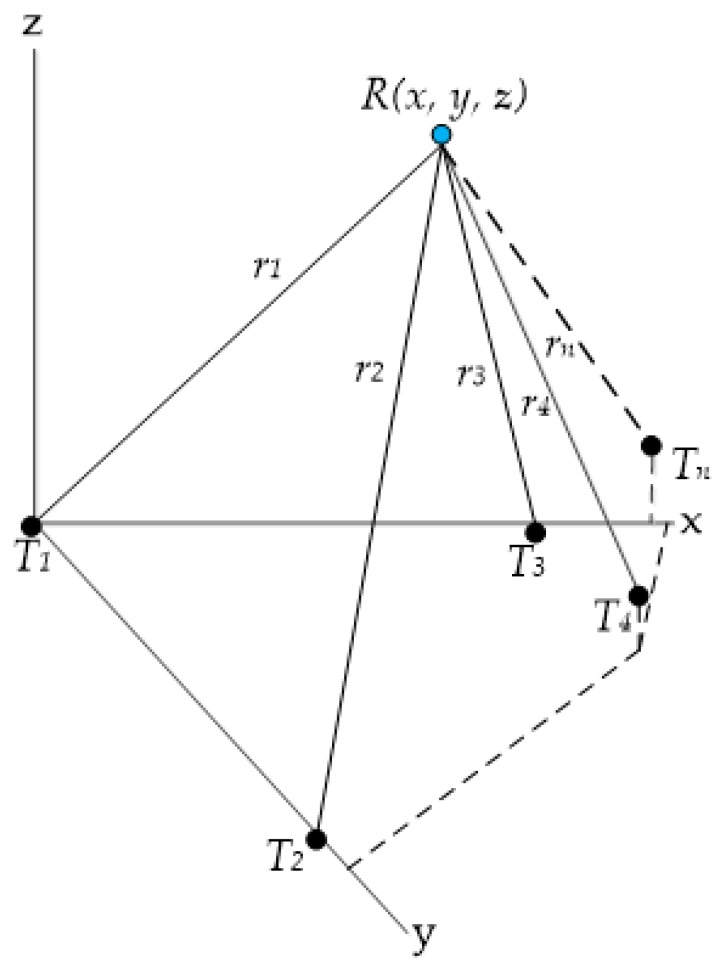
Multistatic sensor arrangement.

In a familiar environment, for example in a building, a multistatic sensor approach can be applied that utilises base stations (BS) at known fixed locations in the test environment. Similar work has been done so far [40], which utilises transmitters at known locations to calculate the mobile station (MS) or receiver coordinates. A platform fitted with an acoustic receiver (R), as shown in Figure 10, can utilise distance measurements from multiple transmitters to calculate its position. An optimised arrangement of transmitters ensures that the Position Dilution of Precision (PDOP), as shown in Equation (58), is kept within an acceptable threshold [41,42,43]. The optimised geometry also results in keeping the cost of the system down:

(58) $$\mathrm{PDOP}=\sqrt{\frac{\sigma_{x}^{2}+\sigma_{y}^{2}+\sigma_{z}^{2}}{\sigma_{R}^{2}}}=\sqrt{\frac{1}{h_{1}^{2}}+\frac{1}{h_{2}^{2}}+\frac{1}{h_{3}^{2}}+\frac{1}{h_{4}^{2}}}$$

where $\sigma_{x}^{2}$, $\sigma_{y}^{2}$_,_ and $\sigma_{z}^{2}$ are the variances of the estimation errors along each axis and $h_{1}$, $h_{2}$, $h_{3}$, and $h_{4}$ are altitudes of the tetrahedron formed by joining unit vectors from four BS and one MS [44]. 

The distance measurements are made based on time-of-flight (TOF) measurements. The general range equation for the multistatic sensor arrangement is:

(59) $$\rho_{k}{}^{t}\left(t_{k}\right)=\left(t_{k}-t^{t}\right)c$$

where $\rho_{k}{}^{t}$ is the actual measurement, $t_{k}$ is the nominal time of the receiver clock *k* at reception, $t^{t}$ is the nominal time of the transmitter clock s at emission, and c is the speed of sound. After taking into consideration the clock biases, propagation delay in the air, multipath error and random measurement noise, the complete expression for range measurement becomes:

(60) $$r_{k}{}^{t}\left(t_{k}\right)=\rho_{k}{}^{t}\left(t_{r,k}\right)-\left(dt_{k}-dt^{t}\right)c+P_{k,t}{}^{t}\left(t_{k}\right)+d_{k,T}{}^{t}\left(t_{k}\right)+d_{t}{}^{t}\left(t_{k}\right)+d_{k,t}\left(t_{k}\right)+\varepsilon_{t}$$

where: $\rho_{k}{}^{t}\left(t_{r,k}\right)$ = geometric distance (m); $dt_{k}$ = receiver clock error (s); $dt^{t}$ = transmitter clock error (s); $P_{k,t}{}^{t}\left(t_{k}\right)$ = propagation delay in air (standard conditions) (m); $d_{k,t}\left(t_{k}\right)$ = error due to hardware code delay at the receiver (m); $d_{t}{}^{t}\left(t_{k}\right)$ = error due to hardware code delay at the transmitter (m); $d_{k,T}{}^{t}\left(t_{k}\right)$ = multipath error (m); $\varepsilon_{t}$ = random measurement noise (m).

The multipath error depends on the relative geometry of the transmitter and the receiver with respect to the surrounding reflective surface as well as its reflective properties. The coordinates of the receiver as well as the timing information are derived from the simultaneous/sequential observation of four (or more) transmitters. Assuming a constant clock error for measurements to all transmitters and neglecting all other error terms, the following system of equations is obtained:

(61) $$r_{k}{}^{n}\left(t\right)=\sqrt{{\left(x^{n}-x_{k}\right)}^{2}+{\left(y^{n}-y_{k}\right)}^{2}+{\left(z^{n}-z_{k}\right)}^{2}}+vdt_{k}\;\left(n=1,\;2,\;3,\;4\right)$$

**Figure 11:**
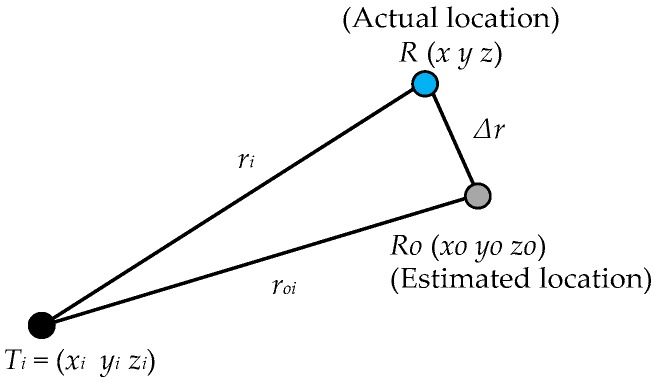
Actual and estimated position of receiver.

Considering this system of equations, a minimum of four BSs are required to yield four equations, which are solved to provide the positioning solution. One approach to solving the time-independent non-linear system of equations is to linearize them by using a recursive least squares algorithm for positioning. With more than four BS being used for calculating the position of the MS, the problem is over-determined and can be solved in the least squares sense to yield an optimal estimate of the MS location. In practice, the solution is obtained iteratively starting from an initial guess of the receiver position ($x_{0}$, $y_{0}$, $z_{0}$) as shown in Figure 11, where:

(62) $$r_{i}^{2}={\left(x-x_{i}\right)}^{2}+{\left(y-y_{i}\right)}^{2}+{\left(z-z_{i}\right)}^{2}$$

(63) $$r_{oi}^{2}={\left(x_{o}-x_{i}\right)}^{2}+{\left(y_{o}-y_{i}\right)}^{2}+{\left(z_{o}-z_{i}\right)}^{2}$$

(64) $$x=x_{0}+\Delta x$$

(65) $$y=y_{0}+\Delta y$$

(66) $$z=z_{0}+\Delta z$$

where Δx, Δy and Δz are the differences between the true solution and the initial guesses (corrections to the nominal values). Equation (62) can also be written as:

(67) $$r_{i}^{2}={\left(\Delta x+x_{o}-x_{i}\right)}^{2}+{\left(\Delta y+y_{o}-y_{i}\right)}^{2}+{\left(\Delta z+z_{o}-z_{i}\right)}^{2}$$

After simplification and disregarding the higher order error terms:

(68) $$\overset{n}{\underset{i=1}{\sum }}r_{i}^{2}-r_{oi}^{2}=\overset{n}{\underset{i=1}{\sum }}2\left[\begin{array}{ccc} x_{o}-x_{i} & y_{o}-y_{i} & z_{o}-z_{i} \end{array}\right]\left\{\begin{array}{c} \Delta x\\ \Delta y\\ \Delta z \end{array}\right\}$$

Equation (68) can be written as:

(69) AΔr=b

where:

(70) $$A=2\left[\begin{array}{c} \begin{array}{ccc} x_{o}-x_{1} & y_{o}-y_{1} & z_{o}-z_{1}\\ x_{o}-x_{2} & y_{o}-y_{2} & z_{o}-z_{2} \end{array}\\ \begin{array}{ccc} \vdots & \vdots & \vdots \\ x_{o}-x_{n} & y_{o}-y_{n} & z_{o}-z_{n} \end{array} \end{array}\right],\Delta r=\left\{\begin{array}{c} \Delta x\\ \Delta y\\ \Delta z \end{array}\right\}\;\mathrm{and}\;b=\left[\begin{array}{c} r_{1}^{2}-r_{01}^{2}\\ r_{2}^{2}-r_{02}^{2}\\ \begin{array}{c} \vdots \\ r_{n}^{2}-r_{0n}^{2} \end{array} \end{array}\right]$$

Denoting the ignored error term by e, Equation (69) can be written as:

(71) e=AΔr−b

or:

(72) $$J=e^{T}e={\left(A\Delta r-b\right)}^{T}A\Delta r-b)$$

This is an unconstrained minimization problem. J needs to be minimized with respect to n unknown values of Δr. Thus:

(73) $$0=\frac{\partial J}{\partial r}=\frac{\partial }{\partial r}\left(\Delta rA^{T}A\Delta r-2b^{T}A\Delta r+b^{T}b\right)$$

(74) $$=2\left(A^{T}A\right)r-2A^{T}b$$

Pre-multiplying Equation (74) by $-1/2{\left(A^{T}A\right)}^{-1}$ yields the least squares solution for linear algebraic equations, which can be written as:

(75) $$\Delta r=A^{**}b$$

where:

(76) $$A^{**}={\left(A^{T}A^{-1}\right)}^{-1}A^{T}$$

$A^{**}$ is called the pseudo inverse of A for overdetermined systems. Note that the order of matrix Δr remains 3 × 1, irrespective of the number of rows in A, as long as there are a minimum of three rows. The number of rows in A denotes the number of BS’s that are in range. To correct the initial guess, a linear (first-order Taylor) expansion of $\rho_{k}$ is adopted. Let n ($x_{n},\;y_{n},\;z_{n}$) denote a preliminary best estimate value. The linearised equation is:

(77) $$-\frac{x_{n}-x^{s}}{R_{n}{}^{s}}\Delta x_{k}-\frac{y_{n}-y^{s}}{R_{n}{}^{s}}\Delta y_{k}-\frac{z_{n}-z^{s}}{R_{n}{}^{s}}\Delta z_{k}+\Delta dt_{k}=\Delta R^{s}$$

where $R_{n}{}^{s}$ is the nominal range measurement to the *s*th BS and $\Delta R^{s}$ is the difference between the actual and nominal range measurements. In addition to the ranging errors that affect the sensor position accuracy, the relative geometry of BS and MS affects the estimation. The linearised equations to s BSs are given by:

(78) $$\left[\begin{array}{c} \Delta x_{k}\\ \Delta y_{k}\\ \begin{array}{c} \Delta z_{k}\\ \Delta dt_{k} \end{array} \end{array}\right]=-{\left(B^{T}\sigma_{0}{}^{2}\;R_{\rho }\;B\right)}^{-1}\;B^{T}\;\sigma_{0}{}^{2}\;R_{\rho }\;\left[\begin{array}{cccc} \Delta R^{1} & \Delta R^{2} & \Delta R^{3} & \begin{array}{cc} \ldots & \Delta R^{s} \end{array} \end{array}\right]$$

where B is the matrix of coefficients of the linear set of equations, $R_{\rho }$ is the covariance matrix of the pseudorange errors, and $\sigma_{0}{}^{2}$ is a scale factor known as the a priori variance of unit weight. 

Various other multilateration algorithms have been proposed in the literature which present numerical algorithms based on iteratively solving simultaneous position equations based on an initial estimate [45,46,47]. A comparison of a linear least squares estimator, a non-linear least squares estimator, and an iteratively reweighted least squares technique was also made [46], where it was shown that the performance of non-linear least squares method was the best. In [48], a probabilistic model of the error in distance measurement for trilateration using Extended Kalman Filtering (EKF) was developed. However, most of the iterative algorithms converge locally, hence are sensitive to the initial estimate. An inappropriate initial estimate can lead to convergence to the wrong local optimum in the vicinity of the initial estimate, described as mirroring in [40]. Besides, iterative algorithms are computationally intensive. Various closed-form trilateration algorithms have also been discussed in the literature [49,50,51,52]. In [53], a closed-form algorithm for solving least squares trilateration problem is discussed, which works for an overdetermined system as well as in cases where there is no real solution. Additionally, global optimization techniques can also be explored, but their computational complexity can render them unsuitable for real-time applications. 

### 5.3. Combination of Multistatic and Monostatic Approaches

**Figure 12:**
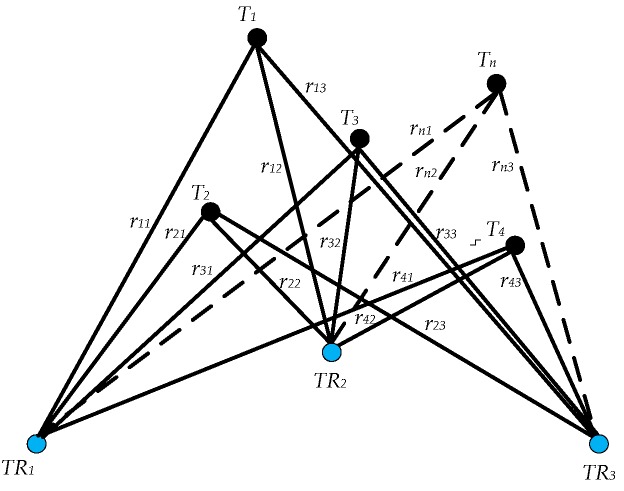
Relative navigation of multiple transceivers.

A third approach combines the monostatic and multistatic approaches. This hybrid approach enables relative navigation among platforms equipped with monostatic transceivers. Swarms of platforms communicate with each other via on-board acoustic sensors to calculate their position as well as augment the knowledge of their environment. The transmitters of the multistatic system are fixed at known locations. The transmitters relay their unique identification information and transmission time along with the acoustic signal to enable positioning based on multilateration. Figure 12 shows the schematic of relative navigation in case of three independent transceivers. $T_{i}$ is the *i*th transmitter fixed at a known location while $TR_{j}$ is the *j*th transceiver on-board the platform, with $r_{ij}$ being the distance between the *i*th transmitter and the *j*th transceiver at a given instant of time.

## 6. Overview of State-of-the-Art Acoustic Sensors

Acoustic sensors, mostly ultrasonic, have been widely used in navigation, especially indoor navigation and personal mobility. In [54], an ultrasonic indoor positioning system based on Time Difference of Arrival (TDOA) is discussed. The proposed system consists of fixed active ultrasonic transmitters and passive receivers arranged in an omnidirectional hexagonal pattern on the mobile platform. The indoor navigation system discussed in [40] is based on Time of Arrival (TOA) to calculate the coordinates of the receiver in real time. Synchronization between the transmitters at fixed known positions on the ceiling and the mobile receiver enables Time Division Multiple Access (TDMA) implementation for positioning of the receiver using multilateration. In [55], a combination of globally and locally-referenced Local Positioning Systems (LPS) is introduced to cover an extensive indoor environment. In addition, certain acoustic LPS employ Direct Sequence Code Division Multiple Access (DS-CDMA) [56] techniques, where the receiver on-board the robot determines its position using TDOA between a reference beacon and other beacons, with each beacon transmitting a unique 255-bit Kasami code. More advanced Code Division Multiple Access (CDMA) based acoustic positioning systems determine TOA at the receiver using signal correlation [57], used in all modern Radio Frequency (RF)-based communication systems, including the GNSS. 

Certain navigation systems like [58,59,60,61] use both RF as well as ultrasonic signals to calculate the position of the mobile platform. Cricket indoor location system [58] uses RF signals for synchronizing the ultrasonic transmitters and receiver on the mobile platform. In Dolphin [59], the relative position of a node placed on the ceiling is calculated by trilateration using TOA of ultrasonic signals from three nodes. The RF signal from the nodes contains node location information as well as time synchronization. A commercial-off-the-shelf (COTS) hardware components based architecture for a self-driving miniature vehicle is developed using real-time operating system (RTOS) in [62]. There 1:10 scale self-driving vehicle has three ultrasonic sensors, three infrared sensors and a camera. In [63], an ultrasonic relative positioning system is developed for a group of ground-based robots. Moving in formation, the robots can determine the distance and orientation of nearby robots based on TOF evaluation of ultrasonic pulses as well as a RF communication link. In [60], personal tracking is achieved with the help of a portable unit called Bat, which consists of a radio transceiver, controlling logic and an ultrasonic transducer. Ultrasound receiver units are placed on the ceiling at known points and are interconnected. The Bats are triggered by a radio message from the base station, which synchronizes the trigger with the receivers. In [61], a multi-block navigation system is developed which utilizes RFID transmitters to trigger relevant beacons to send an ultrasonic signal for localization of the mobile object.

Table 4 lists some representative COTS ultrasonic ranging sensors operating at frequencies ranging from 40 kHz to 500 kHz. It can be observed that as the operating frequency of the ultrasonic ranging sensor increases, the detection range decreases mainly due to higher attenuation. As described in Section 2, bats can vary the frequency of their echolocation calls based on the distance from their prey or an obstruction, which allows them to achieve optimal range and angular resolution performance in various conditions.

Various acoustic navigation aids for visually impaired have been investigated. An ultrasonic FM mobility aid is proposed in [64]. While, in [65], a bio-inspired mobility aid for visually impaired based on echolocation of bats is discussed. This device uses downswept FM ultrasound emissions to detect obstacles, which are perceived as localized sound images corresponding to the direction and size of the obstacles. In [66], a digital signal processor based ultrasonic navigation aid for the visually impaired is presented. The nearest obstacle in front of the user is detected using 2-dimensional echolocation and a binaural audio feedback is provided. GuideCane [67] and UltraCane [68] use ultrasonic sensors in the cane to help visually impaired navigate quickly and safely in the presence of obstacles and hazards. 

## 7. Integration of Acoustic Sensors in Multi-Sensor Navigation Systems

**Figure 13:**
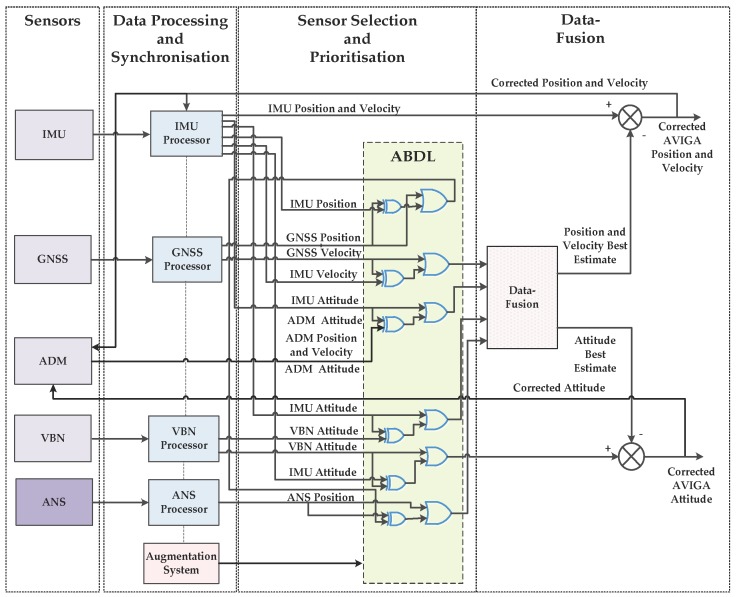
AVIGA architecture.

Currently, air and surface vehicles rely on a combination of GNSS, Inertial Measurement Unit (IMU) and in some cases, Vision-Based Navigation (VBN) and Aircraft Dynamics Model (ADM) virtual sensor for air vehicle navigation and guidance [2]. Integration of ANS (Acoustic Navigation System) with the existing NGS (Navigation and Guidance System) enables accurate and reliable positioning, even in low visibility indoor environments, using low Size, Weight and Power, and Cost (SWaP-C) sensors. GNSSS signals are prone to data degradations or complete loss of signal in dense urban environments as well as indoors due to multipath effects, interference, or antenna obscuration [36,69]. High precision IMUs employ relatively high cost, weight and volume inertial components (e.g., ring laser and fibre optic gyroscopes), rendering them unsuitable for many ground and air vehicles like cars, Remotely Piloted Aircraft Systems (RPAS), etc. Although commercially available Micro Electromechanical Systems (MEMS) devices can support the design of low cost IMUs, their accuracy decreases steeply with operating time. The performance of VBN sensors is affected by low visibility conditions which could be due to night/low light conditions, smoke, fog, precipitation as well as presence of transparent or opaque objects [70]. An ADM virtual sensor is dependent on the aircraft’s physical sensors and is based on the assumption of the aircraft being a rigid body with a constant and static mass distribution [71]. An integration of sensor data achieved through Multi-Sensor Data Fusion algorithms can lead to an improved positioning solution for air and ground platforms. The performance analysis of different multi-sensor integrated architectures, including the Extended Kalman Filter (EKF)-based VBN-IMU-GNSS (EVIG), the EKF-based VBN-IMU-GNSS-ADM (EVIGA) and the Unscented Kalman Filter (UKF)-based VIGA (UVIGA) have shown that these integration schemes provide improvements in position, velocity, and attitude (PVA) data in all flight phases in air vehicles, when compared to individual sensor measurements. In particular, the EVIGA and UVIGA systems achieve horizontal/vertical position accuracies in line with International Civil Aviation Organization (ICAO) CAT-I and CAT-II requirements [71]. Figure 13 presents the NGS architecture including the ANS and thereby resulting in an ANS-VIGA (AVIGA) integration scheme. 

In this integrated system, the data output rate for ANS is 2 Hz, 20 Hz for VBN and GNSS at 2 Hz to augment the MEMS-IMU running at 100 Hz. The IMU position and velocity information are compared to the Global Positioning System (GPS) position and velocity and form the measurement input of the data fusion block. A similar process is also applied to the attitude data, the differences of which are used in the data-fusion algorithms. The data-fusion algorithm provides estimates of the PVA errors, which are then removed from the sensor measurements to obtain the corrected navigation states. The corrected PVA as well as the estimates of the accelerometer and the gyroscope biases are used to update the IMU raw measurements. The attitude data provided by ADM augmentation and by the IMU are compared to feed the data-fusion block at 100 Hz. The attitude data provided by the VBN and IMU sensors are compared at 20 Hz. The best estimate of attitude is compared with the IMU attitude to obtain the corrected attitude. By employing a UKF, the AVIGA system performance in terms of attitude data accuracy can be increased in addition to a significant extension of the ADM validity time. Additionally, a pre-filter can be also used to pre-process the Six Degrees-of-Freedom (6-DoF) ADM navigation solution. Another pre-filter can be employed to process the ANS data to remove any outliers. In order to select the navigation sensors based on its performance (accuracy, availability, continuity, and integrity), a sensor selection and prioritisation approach is employed using Adaptive Boolean Decision Logic (ABDL), as shown in Figure 13.

Multiple operating modes can be implemented using the ABDL, wherein each mode is a unique combination of sensors. The selection of sensors is dictated by the flight phase, integrity alerts (both preventive and reactive) and other factors. Table 5 lists some of the typical characteristics of COTS sensors used for navigation in small sized platforms.

## 8. Conclusions and Recommendations for Future Research

This paper presents acoustic wave propagation and its applications in air and surface vehicles navigation. Taking inspiration from echolocating mammals, especially bats, novel acoustic navigation techniques are introduced. Various sound wave attenuation factors like geometric divergence, atmospheric absorption, ground effect, screening, and wind and temperature gradient effects are discussed in detail. Mathematical error modeling for acoustic range measurements, taking into consideration Doppler shift, multipath and atmospheric effects, is presented. Different acoustic sensor arrangements for navigation, monostatic, multistatic and a combination of the two, are discussed. Also, the state-of-the-art in acoustic sensors and their applications in navigation are presented. Finally, the integration of acoustic sensors in multi-sensor navigation systems is discussed.

The use of acoustic sensors for air and surface vehicle navigation holds very high potential, especially when addressing low SWAP-C requirements. Current research trends indicate that future acoustic sensors will implement sophisticated bio-inspired features, which may significantly enhance their range and resolution performance [75,76]. In future applications, acoustic sources might also be used as Signals of Opportunity (SoOP) for navigation in GNSS challenged/denied environments [77]. Although acoustic sensor networks have been utilized in the design of smart city networks [78] and for intelligent transport systems [79], their potential has not yet been fully exploited in air and surface navigation systems. So, acoustic sensors are expected to attract much research and industry efforts in the near future, due to a growing interest in alternative sources of navigation data in urban/indoor environments for a variety of civil and military applications. In order to undertake the development of either monostatic or multistatic acoustic sensors, a careful analysis of aero-acoustic effects is required, especially for aviation applications. In particular, it is essential to assess the magnitude of potential in-band and out-of-band interferences (e.g., engine, propellers, aerodynamic noise) and implement appropriate engineering solutions that mitigate/prevent the negative effects of such interferences on sensor performance. For instance, the use of larger propellers in rotary-wing aircraft might significantly reduce in-band interferences for acoustic sensors operating at higher frequencies. Similar considerations apply to the acoustic emissions produced by gas-turbine engines (i.e., size and number of rotor blades). In this case, however, noise reduction and frequency tailoring can be obtained by adopting other engineering solutions, such as chevrons, liners and noise absorptive materials [80].

## Figures and Tables

**Table 1:** Echolocation call types for different bat species based on diet [11,12].

Diet	Echolocation Call Type	Bat Species
Fruits	Broadband clicks of short duration	Egyptian fruit bat
Moths, beetles, flies and other insects	Narrowband with dominant fundamental harmonic	Eastern red bat
Flying insects and small fruits	Multiharmonic narrowband, faintly audible to humans	Black-bearded tomb bat
Aquatic insects like midges, crane flies and black flies	Short, broadband, with dominant fundamental harmonic	Daubenton’s bat
Large insects, spiders and small vertebrates	Short, multiharmonic broadband	Greater false vampire bat
Moths	Long, multiharmonic broadband	Madagascar sucker-footed bat
Butterfly, moths and beetles	Constant frequency (CF) & Frequency Modulated (FM)	Greater horseshoe bat
Beetles, moths, flies, wasps, and flying ants	Downswept FM narrowband	Big brown bat
Beetles, moths, flies, and small insects	FM broadband	Townsend’s big-eared bat

**Table 2:** Acoustic sensor ranging parameters.

Type	Parameters
Design parameters	Transmitted power, carrier frequency and PRF
Measured observables	Range, velocity, azimuth and elevation
Environmental parameters	Temperature, wind, humidity and environmental layout
Performance indicators	Position accuracy and maximum range

**Table 3:** Ranging parameters.

Variable	Value (Unit)
Speed of sound at sea level ($c_{0}$)	340.27 (m/s)
Time of flight (t)	0.009 (s)
Mach number for the sound source (M)	0.024
Direction of receiver motion to the LOS (θ)	30 (deg)
Variation of temperature with height (λ)	−0.0065 (K/m)
Speed of sound emitted by source (c) at 20 °C	343 (m/s)
Distance between *i*th transmitter and receiver ($\left|Tr_{i}-R_{i}\right|$)	10 (m)
Distance between *i*th transmitter and reflection point ($\left|Tr_{i}-S_{i}\right|$)	2 (m)
Distance between *i*th receiver and reflection point ($\left|R_{i}-S_{i}\right|$)	8.328 (m)
Sea-level temperature ($T_{0}$)	288 (K)
Horizontal wind velocity ($v_{w}$)	2.95 (m/s)
Angle of wavefront normal with the horizontal (δ)	30 (deg)

**Table 4:** Commercially available ultrasonic ranging sensors.

Ultrasonic Sensor	Manufacturer	Transducer Frequency	Detection Range (mm)
MA40SR/S	Murata	40 kHz	Sound Pressure Level (SPL) dependent
MB8450	MaxBotix	42 kHz	500–5000
MA58MF14-7N	Murata	58 kHz	SPL dependent
UC6000-30GM-E6R2-V15	Pepperl + Fuchs	65 kHz	350–6000
XX630A3PCM12	Telemecanique Sensors	75 kHz	203–8000
3RG6014-3AD00-PF	Pepperl + Fuchs	80 kHz	600–6000
UC4000-30GM-IUR2-V15	Pepperl + Fuchs	85 kHz	200–4000
UM30-214113	Sick	120 kHz	350–3400
UB2000-F54-I-V15	Pepperl + Fuchs	175 kHz	80–2000
UC2000-30GM-IUR2-V15	Pepperl + Fuchs	180 kHz	80–2000
BUS M18M1-GPXI-12/100-S92G	Balluff	200 kHz	120–1300
T30UIPAQ	Banner	228 kHz	150–1000
UGT507	ifm electronic	230 kHz	Maximum of 1200
UNDK 30U6103/S14	Baumer	240 kHz	100–1000
UNDK 20U 6912	Baumer	290 kHz	60–400
XX518A3PAM12	Telemecanique Sensors	300 kHz	51–508
UB400-12GM-E5-V1	Pepperl + Fuchs	310 kHz	30–400
BUS M30M1-PPX-03/25-S92K	Balluff	320 kHz	30–350
XXV18B1PBM12	Telemecanique Sensors	360 kHz	3–50
UB500-18GM75-E5-V15	Pepperl + Fuchs	380 kHz	30–500
UB300-18GM40-E5-V1	Pepperl + Fuchs	390 kHz	30–300
UM30-212113	Sick	400 kHz	60–350
XX512A1KAM8	Telemecanique Sensors	500 kHz	25–152

**Table 5:** Transport grade COTS Navigation sensor characteristics [72,73,74].

Sensor	Data Output Rate (Hz)	Size (L×W×H) ($cm^{3}$)	Weight (g)	Power (W)
MEMS based Inertial Navigaiton System	100	12	15–50	<0.5
Vision Based Navigation	20	64	50–100	~1
GNSS receiver	2	10	20–60	<0.4
